# Incomplete B-cell reconstitution in ART-treated people living with HIV is associated with EBV-linked lymphoma progression

**DOI:** 10.3389/fimmu.2026.1791480

**Published:** 2026-03-18

**Authors:** Niyireth Peñaloza, Laura Rubio, Tito A. Sandoval, Ricardo Ballesteros-Ramírez, Alvaro Cadena, Sandra Milena Gualtero, Sandra Liliana Valderrama-Beltrán, Janeth Salazar-Vargas, Fabian Mejía, Adriana Cuéllar, Sandra Quijano

**Affiliations:** 1 Grupo de Inmunobiología y Biología Celular, Pontificia Universidad Javeriana, Bogotá, Colombia; 2 Grupo de Investigación en Ciencias del Laboratorio Clínico, Hospital Universitario San Ignacio, Pontificia Universidad Javeriana, Bogotá, Colombia; 3 Department of Radiation Oncology, Washington University School of Medicine, Louis, MO, United States; 4 Grupo de Oncología PUJ-HUSI, Facultad de Medicina, Pontificia Universidad Javeriana, Bogotá, Colombia; 5 Centro Javeriano de Oncología, Hospital Universitario San Ignacio, Bogotá, Colombia; 6 Servicio de Hematología, Hospital Universitario San Ignacio, Bogotá, Colombia; 7 Pontificia Universidad Javeriana, Unidad de Infectología, Departamento de Medicina Interna, Facultad de Medicina, Bogotá, Colombia; 8 Hospital Universitario San Ignacio, División de Enfermedades Infecciosas, Departamento de Medicina Interna, Grupo de Investigación en Enfermedades Infecciosas, Bogotá, Colombia; 9 Servicio de Patología, Hospital Universitario San Ignacio, Bogotá, Colombia

**Keywords:** antiretroviral therapy, B lymphocytes, chronic inflammation, Epstein–Barr virus, HIV, immune reconstitution

## Abstract

**Background:**

Despite the widespread use of antiretroviral therapy (ART), people living with HIV continue to exhibit persistent immune alterations. Among the most affected cell populations are B lymphocytes, which show disruptions in differentiation, class-switch recombination, and the development of immunological memory. However, the relationship between these abnormalities, Epstein–Barr virus (EBV) coinfection, and clinical outcomes across different stages of HIV disease remains poorly understood.

**Methods:**

We analyzed 272 patients living with HIV from Hospital Universitario San Ignacio (Bogotá, Colombia), divided in four groups: clinical stage (S1, S2, S3 and lymphoma), clinical categories: AIDS-defining diseases, non-AIDS-defining diseases, and coinfections (CI), time on ART (<1 year, >1 year); and EBV coinfection. B lymphocyte subpopulations and immunoglobulin isotypes were quantified (next-generation flow cytometry). Cytokines (multiplex assays) and EBV viral load (quantitative PCR) were measured.

**Results:**

HIV progression was observed, associated with B cell compartment remodeling, characterized by depletion of naïve and memory B cells (smIgA1–2 and smIgG1–4) and an increase in immature/transitional cells and alterations in smIgM with isotype switching. These variations correlated negatively with clinical stage (ρ up to –0·43). The cytokine profile showed a persistent inflammatory signature (MIP-1β, G-CSF, FLT-3L, and IL-3). EBV coinfection intensified this phenotype, being associated with elevated levels of IL-6, IL-10, IL-15, TNF-α, and sCD40L, and with greater loss of memory cell subpopulations. Patients with AIDS-defining diseases and lymphomas exhibited the most profound alteration. Even after prolonged ART (>1 year), B cell reconstitution remained incomplete and biased toward immature phenotypes.

**Conclusions:**

This study shows that recovery of the B cell compartment under ART is incomplete and functionally unbalanced. Humoral memory loss, bias toward immature phenotypes, and persistent inflammation create a state of dysregulation that is exacerbated by EBV coinfection, especially in advanced clinical stages. These immunological defects provide a basis for the pathogenesis of non-AIDS-defining diseases and Epstein-Barr-associated lymphomas. Our findings support the need to integrate advanced immunological assessments, including standardized flow cytometry, as a complement to CD4+ count and viral load, in order to more accurately characterize immune competence and anticipate clinical risks in people living with HIV.

## 1 Introduction

Human immunodeficiency virus (HIV) infection remains a major global public health challenge, with an estimated 40.8 million people currently living with the virus and 1.3 million new cases reported worldwide in 2024, according to the World Health Organization (WHO) (1).

The introduction and widespread use of ART have transformed HIV infection, markedly lowering mortality and improving quality of life for PLHIV. However, the health burden remains considerable: AIDS-defining diseases (ADD) still contribute to mortality, while non-AIDS-defining diseases (NADD), including non-AIDS-related cancers, cardiovascular disease, and metabolic complications, have become leading drivers of morbidity and mortality (2).

Despite effective virologic suppression, PLHIV often remain in a state of chronic immune activation marked by systemic inflammation and sustained production of proinflammatory cytokines, resembling a low-grade but persistent “cytokine storm.” This inflammatory milieu promotes T- and B-cell exhaustion and is further amplified by gut microbiota dysbiosis and microbial translocation (3). Consequently, both AIDS-defining and non-AIDS-defining comorbidities remain prevalent and contribute significantly to disease burden (4). HIV infection also profoundly disrupts B-cell homeostasis, including expansion of immature/transitional B cells (I/T-BL) that correlates with CD4+ lymphopenia and impaired early B-cell maturation, potentially influenced by compensatory IL-7 signaling (5). In parallel, HIV is associated with increased exhausted and hyperactivated mature B cells and an expansion of short-lived plasmablasts, consistent with polyclonal activation and defective humoral maturation (6).

Longitudinal studies indicate that ART initiation only partially restores B-lymphocytes subsets. Notably, I/T-BL frequencies remain elevated compared with healthy donors, underscoring the incomplete reconstitution of B-lymphocytes ontogeny (7). Moreover, I/T-BL accumulates in peripheral blood (PB) and exhibit defective migration to lymphoid follicles, impairing their integration into germinal centers (GC) and sustaining an immature and dysfunctional immune phenotype (8).

A major consequence of HIV-associated B-cell dysfunction is germinal center (GC) damage, which impairs B-cell maturation, memory B-cell generation, and production of high-affinity antibodies. GC integrity depends on coordinated interactions between B cells and follicular helper T cells (Tfh), particularly through CD40-CD40L signaling (9, 10). In HIV infection, Tfh and B cells may expand within follicles, but their function is compromised, leading to defective maturation and progressive loss of humoral memory (9, 10). HIV also drives the accumulation of atypical, often T-bet-expressing B-cell populations outside GCs, reflecting defects in clonal selection, somatic hypermutation, and class-switch recombination that ultimately limit protective antibody responses (11).

These immune alterations are exacerbated by coinfection with oncogenic viruses such as Epstein-Barr virus (EBV). Impaired T-lymphocytes surveillance enables EBV persistence and reactivation, fueling extrafollicular B-lymphocytes maturation and the production of low-affinity antibodies (12, 13). In this setting, I/T-BL not only accumulates as markers of GC dysregulation but also serves as reservoirs for EBV, perpetuating chronic infection. This compromised environment fosters aberrant B-lymphocytes expansion and increases susceptibility to EBV-associated lymphomas, which remain disproportionately frequent and carry poor prognosis in PLHIV (14).

Standardized assessment of HIV-associated B-cell alterations is challenging in clinical practice. The EuroFlow Consortium developed a widely used multicolor B-lymphocyte panel that resolves B-cell maturation stages while assessing surface immunoglobulin isotypes, enabling high-resolution detection of differentiation defects and supporting diagnosis of primary immunodeficiencies. Its application to HIV may improve monitoring of B-cell dysregulation and related complications (15).

This study aimed to use EuroFlow panels to comprehensively characterize alterations in peripheral B-lymphocytes subpopulations across clinical stages of HIV infection in people under ART, and to determine their relationship with EBV coinfection, systemic cytokine profiles, and clinical outcomes. By integrating immunophenotyping, cytokine quantification, and viral load analysis, we sought to identify immunological patterns that may underline incomplete immune reconstitution and increased susceptibility to HIV-associated comorbidities.

## 2 Material and methods

### 2.1 Study population and blood samples

Peripheral blood (PB) samples anticoagulated with EDTA were collected from 272 patients diagnosed with HIV infection, attended between March 2023 and April 2025 at the Infectious Illness and Hematology Services of the Hospital Universitario San Ignacio (HUSI) in Bogotá, Colombia.

Most samples were processed on the same day of collection or within a maximum period of 36 hours; peripheral blood cells were isolated and analyzed from fresh samples without cryopreservation. The samples were then analyzed using a hematology analyzer (BC-6200, Mindray) for complete blood count parameters. During Flow cytometry analysis, although viability marker was not included, cell populations and sample quality were assessed based on forward and side scatter (FSC and SSC) parameters, allowing the exclusion of debris, together with CD45 expression for identification of leukocyte populations.

Cohort stratification was performed considering clinically relevant variables for prognosis and disease progression, based on a comprehensive evaluation of clinical and paraclinical criteria: (i) clinical stage (S1-S3) of HIV infection according to the Centers for Disease Control and Prevention (CDC) classification, used to reflect the classical progression of infection; (ii) clinical categories: the presence of coinfections (CI); the existence of AIDS-defining diseases, indicative of advanced immunosuppression; and non-AIDS-defining diseases, considered comorbidities. Additionally, ART duration (<1 year *vs*. >1 year) was included to evaluate the influence of treatment length on immune reconstitution. As a comparative group, 20 healthy controls without HIV diagnosis were included. A detailed description of these categories is presented in **Supplementary Tables 1**-**5**.

All participants underwent a complete blood count using the Coulter DXH900 hematology analyzer at the HUSI Clinical Laboratory, and clinical data were obtained from institutional electronic health records.

Adults (≥18 years) with confirmed HIV on ART who provided written informed consent were included. The study was approved by the Ethics Committees of Pontificia Universidad Javeriana and HUSI (FM-CIE-0778-19, FM-CIE-1103-23, FM-CIE-0743-25) and conducted in accordance with Colombian Ministry of Health Resolution 8430 (1993) and the Declaration of Helsinki. Exclusion criteria were pregnancy, active chemotherapy, and cognitive impairment precluding consent.

### 2.2 Plasma cytokine quantification

Plasma cytokine and chemokine levels were quantified using a high-sensitivity multiplex immunoassay (Milliplex^®^ MAP, customized kit #HCYTA-60K, Millipore). Determinations were performed on plasma samples from 217 PLHIV and 20 healthy controls, following the manufacturer’s protocol.

The assay was conducted on a Luminex platform, with data acquisition via xPONENT software and subsequent analysis with Belysa. The analyte panel included mediators involved in dysregulated immune activation associated with chronic HIV and Epstein-Barr virus (EBV) infection: IL-1α, IL-1β, IL-1Ra, IL-2, IL-3, IL-4, IL-5, IL-6, IL-7, IL-8, IL-10, IL-12 (p40), IL-12 (p70), IL-15, IL-18, IL-22, TNF-α, IFN-α2, IFN-γ, sCD40L, FLT-3L, EGF, FGF, G-CSF, GM-CSF, MIP-1α/CCL3, and MIP-1β/CCL4 (16).

### 2.3 EuroFlow-based next-generation flow cytometry immunophenotyping of B-cell subsets and immunoglobulin isotypes

PB samples were processed to identify and quantify B-lymphocytes subpopulations by next-generation flow cytometry, following standardized protocols of the EuroFlow Consortium (15). The complete bulk lysis protocol (BulkLysis, Catalog No.555899) was applied to 10 mL of EDTA-anticoagulated PB, adjusting the final concentration to 1x10^6^ to 1x10^7^ cells per tube for immunophenotypic analysis.

B-lymphocyte subpopulations were characterized using two monoclonal antibody panels of 12 and 8 fluorochromes, respectively (detailed in **Supplementary Tables 7**, **8**). The 12-color panel was used for detailed phenotypic characterization of B-cell subpopulations, whereas the 8-color panel corresponded to the EuroFlow Lymphocyte Screening Tube (LST), which was used to assess immunoglobulin light chain (kappa and lambda) expression to evaluate B-cell clonality and complement B-cell compartment characterization. Stained samples were acquired on a FACSLyric™ flow cytometer (BD Biosciences) and analyzed with Infinicyt software (Cytognos SL, CYT-INFINICYT). Instrument calibration and compensation followed EuroFlow guidelines, with daily performance checks using CS&T calibration beads (BD Biosciences), ensuring automated voltage adjustment and signal stability (17).

Data analysis followed the gating strategy defined in EuroFlow protocols (**Supplementary Figure 1**), allowing precise discrimination and quantification of B-lymphocytes subpopulations. The evaluated populations included: B- lymphocytes (CD19^+^ selection); immature/transitional B- lymphocytes (I/T-BL; CD5^+^het/CD38^+^hi/CD24^+^hi/CD27^+^/smIgM^++^/IgD^+^); naïve B- lymphocytes (CD5^-^/CD27^-^/CD38^+^d/CD24^+^het/smIgM^+^/smIgD^++^); unswitched memory B- lymphocytes (USMBCs; CD5^-^/CD27^+^/CD38^-^/smIgM^+^/smIgD^++^); switched memory B- lymphocytes (SMBCs; CD5^-^/CD27^+/-^/CD38^-^/smIgM^-^/smIgD^-^); and surface immunoglobulin isotypes (smIgH: smIgM, smIgA1, smIgA2, smIgG1, smIgG2, smIgG3, smIgG4).

### 2.4 EBV viral load quantification in plasma

Viral DNA was extracted from 400 μL of plasma using the automated MagCore^®^ Plus II system (RBC Bioscience) and the MagCore^®^ Viral Nucleic Acid Extraction Kit (high sensitivity). Extraction was reinforced by adding RNA Carrier, Proteinase K, and internal beta-globin control.

EBV quantification was performed by real-time PCR on a C1000 Touch thermal cycler, using EBV Real Time Mix master mix (AB Analitica, RealQuality RQ-EBV). Amplification followed the manufacturer’s protocol and was optimized for high sensitivity and specificity.

### 2.5 Statistical analysis

Non-parametric statistical tests were used to assess group differences. First, a Kruskal-Wallis global test compared distributions across groups. When significant results were obtained (p < 0.05), pairwise comparisons were performed using the Wilcoxon rank-sum test with Bonferroni correction to control type I error risk from multiple testing. Exact p-values are shown for significant comparisons.

## 3 Results

### 3.1 Description of the study population

A total of 272 PLHIV on ART were included and stratified by clinical stage (S1, S2, S3), with a separate subgroup of *de novo* lymphoma cases (**Table 1**), as defined by standardized diagnostic and therapeutic follow-up criteria (18).

**Table 1:** Clinical and demographic characteristics of people living with HIV stratified by clinical stage.

Variables	Stage 1	Stage 2	Stage 3	Lymphoma	*P-*value
	(n = 72)	(n = 100)	(n = 92)	(n = 8)	
Age, median (IQR), years	37 (28-45.75)	33.52 (26-39)	38.47 (30-44)	47.5 (38-57.75)	<0.001
Gender, n (%)					ns
- Male	68 (94.4)	98 (98)	82 (89.1)	8 (100)	
- Female	4 (5.6)	2 (2)	10 (10.9)	0 (0)	
Sexual orientation, n (%):					ns
- MSM	50 (69.4)	71 (71)	46 (50)	6 (71.4)	
- MSW	11 (15.3)	16 (16)	20 (21.7)	0 (0)	
- Heterosexual	6 (8.3)	4 (4)	16 (17.4)	1 (14.3)	
- Bisexual	5 (7)	9 (9)	10 (10.9)	1 (14.3)	
Time since diagnosis, years	6.6 (2.8-10.3)	4.9 (0-24.6)	3.9 (0-32.7)	5.9 (0-32.75)	
ART adherence, n (%):					ns
- Good >95%	61 (84.7)	84 (84)	70 (76.1)	5 (62.5)	
- Poor <95%	4 (5.6)	11 (11)	11 (11.95)	1 (12.5)	
- Not specified	7 (9.7)	5 (5)	11 (11.95)	2 (25)	
Time on ART, years	6.5 (3-10)	4.5 (0-21.7)	5.3 (0-21.6)	5.34 (0-21.6)	ns
<1 year on ART, n	9	18	17	5	0.008
Last HIV viral load, n (%):					0.003
- Detectable	3 (4.2)	9 (9)	17 (18.47)	3 (37.5)	
- Undetectable	67 (93.1)	86 (86)	69 (75)	5 (62.5)	
- Not specified	2 (2.7)	5 (5)	6 (6.53)	0 (0)	
HIV viral load categories, n (%):					ns
- 51–200 copies/mL	2 (66.6)	5 (55.5)	7 (41.2)	0 (0)	
- >200–1000 copies/mL	1 (33.4)	1 (11.2)	2 (11.7)	0 (0)	
- >1000 copies/mL	0 (0)	3 (33.3)	8 (47.1)	3 (100)	
Current CD4+ T lymphocytes, mean (SD), cells/µL	834.57 (286.78)	649.96 (206.93)	387.74 (249.22)	361.38 (479.17)	<0.001
Current CD8+ T lymphocytes, mean (SD), cells/µL	748.46 (356.36)	698.12 (296.68)	743.34 (340.29)	686.74 (542.44)	ns
Current CD4/CD8 ratio, mean (SD)	1.12 (0.63)	1.17 (1.34)	0.57 (0.35)	0.43 (0.30)	<0.001
CD4+ T lymphocytes at diagnosis, mean (SD), cells/µL	612 (1941.07)	355.71 (104.70)	155.66 (142.54)	321 (418.03)	<0.001
CD4/CD8 ratio at diagnosis, mean (SD)	0.78 (63.6)	2.9 (9.9)	1.11 (4.16)	0.37 (0.21)	<0.001
EBV viral load status, n (%):					<0.001
- Detectable	2 (10.8)	6 (6)	10 (10.9)	8 (100)	
- Undetectable	58 (89.2)	94 (94)	82 (89.1)	0 (0)	
Lymphoma type, n (%):	NA	NA	NA	8 (100)	NA
- Burkitt (BLT)	–	–	–	1 (12.5)	
- Diffuse Large B-Cell Lymphoma (DLBCL)	–	–	–	2 (25)	
- Plasmablastic (PL)	–	–	–	2 (25)	
- Hodgkin (HL)	–	–	–	3 (37.5)	

NA, not applicable; ns, not significant; ND, no data; SD, standard deviation; MSM, men who have sex with men; MSW, men who have sex with women; ART, antiretroviral therapy; EBV, Epstein-Barr virus; BLT, Burkitt lymphoma; PL, plasmablastic lymphoma; DLBCL, diffuse large B-cell lymphoma; HL, Hodgkin lymphoma. Statistically significant differences between stages are indicated as exact p-values, using the chi-square test for categorical variables or the Kruskal-Walli’s test for comparisons of cell populations.

Patients with lymphoma were significantly older (median 47.5 years, IQR 11.66) than those in stages 1 and 2 (p= 0.001), consistent with the recognized association between advanced age and increased incidence of lymphoma, while sex distribution and sexual orientation (MSM *vs* heterosexual) did not differ significantly across groups. Time since HIV diagnosis was longest in the lymphoma group (median 5.9 years). ART duration also varied (p= 0.008), being shortest in S1 (median 3.6 years) and longest in lymphoma cases (median 5.34 years).

Regarding virological control, detectable HIV viremia was observed in 37.5% of the lymphoma group versus only 9% in S2 (p= 0.003), suggesting greater viral persistence in lymphoma. Furthermore, CD4^+^ T-lymphocytes counts at diagnosis declined progressively with higher clinical stage (p< 0.0001), reflecting cumulative immunosuppression.

As expected, the lymphoma cohort encompassed aggressive subtypes commonly associated with immunodeficiency: 12.5% Burkitt lymphoma, 25% diffuse large B-cell lymphoma (DLBCL), 25% plasmablastic lymphoma, and 37.5% Hodgkin lymphoma, a spectrum consistent with HIV-associated lymphomas (17).

Because Epstein-Barr virus (EBV) is an established driver of lymphomagenesis in immunocompromised settings (19), we quantified EBV viral load in all participants. Strikingly, 100% of lymphoma patients had detectable EBV viremia compared to 10.8% (S1), 6% (S2), and 10.9% (S3) (p < 0.001). These data strongly implicate EBV reactivation in lymphoma onset among PLHIV. The co-occurrence of immunosuppression, HIV viremia, and EBV activation underscores the immunovirological interplay underpinning HIV-associated hematologic malignancies (20).

Although ART has markedly reduced AIDS-related mortality, non-AIDS-defining diseases have emerged as principal causes of morbidity and death in PLHIV, driven by persistent inflammation and immune activation (18). Consistent with this, we observed a stepwise increase in the prevalence of non-AIDS-defining diseases across advancing HIV stages.

In our study, we observed a statistically significant difference in the frequency of total plasmablasts and their subpopulations (kappa and lambda) in patients with EBV coinfection compared with controls (p = 0.01; **Supplementary Table 16**). However, no statistically significant differences were identified across the other clinical categories evaluated, including clinical stage, presence of ADD, NADD or coinfection (CI), or duration of antiretroviral therapy.

Analysis of risk factors (**Table 2**) revealed significant differences between groups. Recreational drug use decreased progressively with advancing clinical stage (p= 0.04).

**Table 2:** Risk factors and immunovirological characteristics of people living with HIV stratified by clinical stage.

Variables	Stage 1 (n = 72)	Stage 2 (n = 72)	Stage 3 (n = 92)	Lymphoma (n = 8)	*p-*value
Smoking, n (%):					ns
- Yes	18 (25)	30 (30)	19 (20.7)	2 (25)	
- No	54 (75)	70 (70)	73 (79.3)	6 (75)	
Recreational drug use, n (%):					0.04
- Yes	11 (15.3)	23 (23)	9 (9.9)	3 (37.5)	
- No	61 (84.7)	77 (77)	82 (90.1)	5 (62.5)	
- Not specified	0 (0)	0 (0)	1 (1.1)	0 (0)	
Substance use disorder, n (%):					ns
- Yes	5 (7)	7 (7)	5 (4.4)	0 (0)	
- No	67 (93)	93 (93)	86 (94.5)	8 (100)	
- Not specified	0 (0)	0 (0)	1 (1.1)	0 (0)	
Chemex, n (%):					0.005
- Yes	2 (2.7)	1 (1)	2 (2.2)	0 (0)	
- No	40 (55.6)	34 (34)	27 (29.3)	6 (75)	
- Not specified	30 (41.7)	65 (65)	63 (68.5)	2 (25)	
History of virologic failure, n (%):					0.014
- Yes	1 (1.6)	1 (1)	8 (8.6)	1 (12.5)	
- No	63 (87.5)	94 (94)	75 (81.5)	5 (62.5)	
- Not specified	8 (5.6)	5 (5)	9 (9.9)	2 (25)	
Currently in virologic failure, n (%):					<0.001
- Yes	0 (0)	0 (0)	0 (0)	1 (12.5)	
- No	65 (90.3)	96 (96)	84 (91.3)	5 (62.5)	
- Not specified	7 (9.7)	4 (4)	7 (8.7)	2 (25)	
Currently in immunologic failure, n (%):					ns
- Yes	0 (0)	0 (0)	4 (4.3)	0 (0)	
- No	64 (88.9)	96 (96)	82 (89.1)	5 (62.5)	
- Not specified	8 (11.1)	4 (4)	6 (6.6)	3 (37.5)	
PLHIV without AIDS-defining diseases, non-AIDS-defining diseases, or coinfections (CI), n (%):					ns
- Yes	12 (16.7)	13 (13)	10 (10.9)	0 (0)	
- No	60 (33.3)	87 (87)	82 (89.1)	8 (100)	
CI, n (%):					ns
- Yes	47 (65.3)	78 (78)	65 (70.7)	4 (50)	
- No	24 (33.3)	22 (22)	27 (29.3)	4 (50)	
- Not specified	1 (1.4)	0 (0)	0 (0)	0 (0)	
AIDS-defining diseases, n (%):					<0.001
- Yes	0 (0)	0 (0)	39 (70.7)	6 (50)	
- No	72 (100)	100 (100)	53 (29.3)	2 (50)	
Non-AIDS-defining diseases, n (%):					ns
- Yes	32 (44.4)	26 (26)	34 (36.9)	3 (37.5)	
- No	40 (55.6)	74 (74)	58 (63.1)	5 (62.5)	

ns, not significant; ND, no data. Statistically significant differences between stages are indicated with asterisks (*p < 0.05, **p < 0.01, ***p < 0.001), using the chi-square test. PLHIV without AIDS-defining diseases, non-AIDS-defining diseases, or coinfections (CI).

Treatment failure patterns also differed: virologic failure (if on two consecutive occasions you have > 200 copies/mL after 24 weeks of ART) was significantly more frequent in Stage 3 compared with Stages 1 and 2 and the lymphoma subgroup (p= 0.0014). In contrast, immunologic failure rates did not differ significantly between groups (p= ns), although sporadic cases were observed in advanced stages and among patients with lymphoma.

### 3.2 B-lymphocytes alterations across clinical stages of HIV and their association with lymphoma

**Figure 1:**
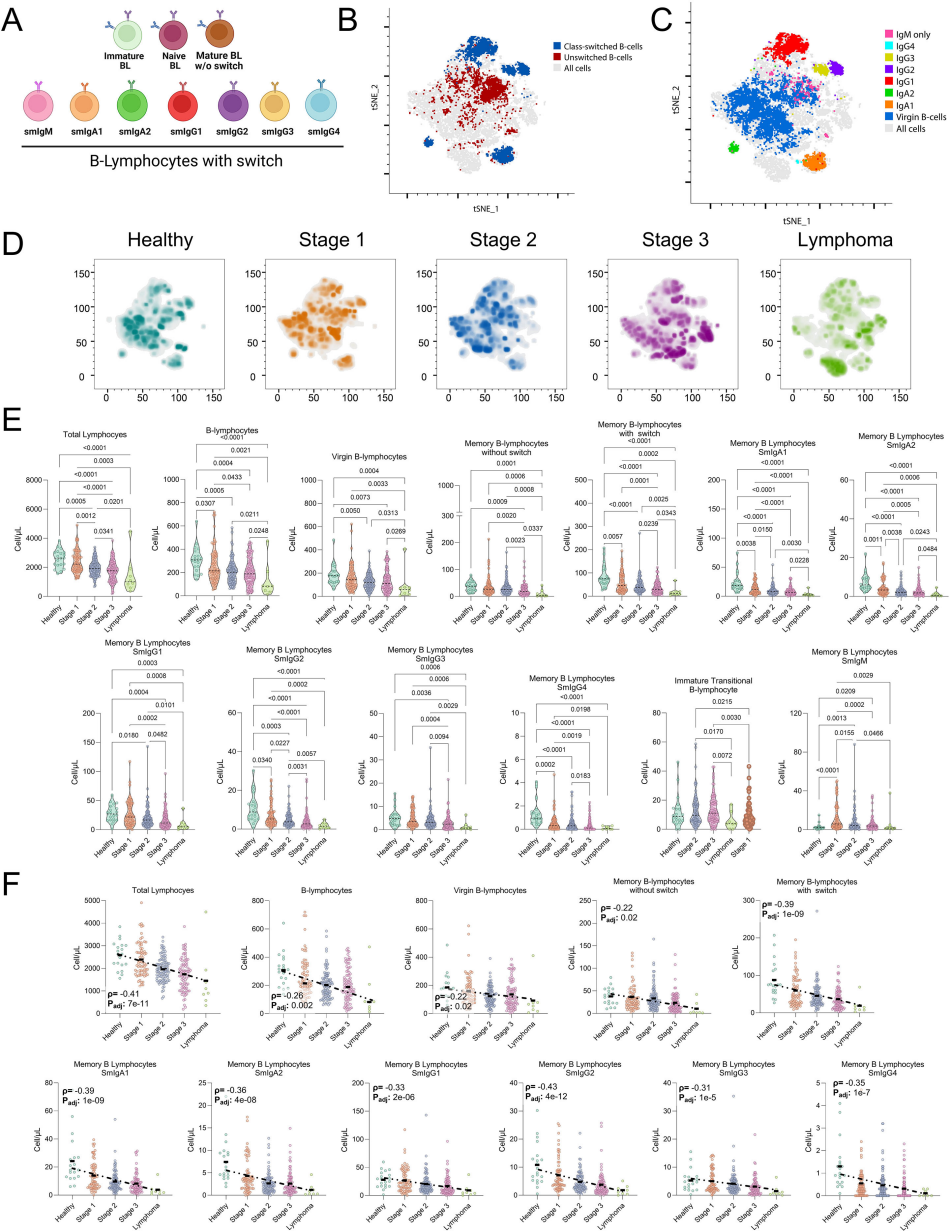
Progressive alterations in B-lymphocytes subpopulations in people living with HIV: association with clinical stages, lymphoma presence, and disease progression. **(A)** Schematic representation of the main B-lymphocytes subpopulations. Shown are I/T-BL, naïve B lymphocytes, and memory B-lymphocytes (MBCs), further differentiated into USMBCs and SMBCs, as well as surface isotypes (smIgM, smIgA1-2, and smIgG1-4). This schematic illustrates the classification strategy used for phenotypic analysis. **(B-E)** Distribution of B-lymphocytes in peripheral blood (PB) by dimensionality reduction. **(B)** Density map showing the global distribution of B-lymphocytes populations across all analyzed samples. **(C)** Identification of specific subpopulations highlighting USMBCs, SMBCs, and I/T-BL. **(D)** Differentiation of each B-lymphocytes subpopulation according to its immunophenotypic profile. **(E)** Comparison of B-lymphocytes distribution in healthy controls, PLHIV at clinical stages 1, 2, and 3, and patients with HIV-associated lymphoma, showing variations in population composition and density throughout clinical progression. **(F)** Progressive decline in absolute counts of total B lymphocytes, naïve B lymphocytes, and MBCs in PLHIV stratified by clinical stage (S1, S2 and S3), in patients with lymphoma, and in healthy controls. A significant reduction was observed in total B lymphocytes, naïve B lymphocytes, and MBCs (USMBCs and SMBCs). In particular, smIgA1–2 and smIgG1–4 isotypes showed a marked decline, most pronounced in lymphoma patients. In contrast, significant expansion was observed in subpopulations such as I/T-BL and SMBC−/smIgM+. Violin plots display the median, dispersion, and density of each group. Correlation plots show absolute counts of different B-lymphocytes subpopulations (cells/μL) in healthy controls (lime green), S1 (orange), S2 (sky blue), S3 (lavender), and HIV-associated lymphoma patients (olive green). The Y-axis represents absolute cell counts, and the X-axis represents the clinical groups compared. Dashed lines indicate linear correlation trends; Spearman’s correlation coefficient (ρ) and adjusted p-value are shown in each panel. Statistically significant differences between stages are indicated.

Based on the initial cohort characterization, we next evaluated immunophenotypic alterations in B-lymphocytes subpopulations in PLHIV, stratified by clinical stage (S1-S3) and the presence of lymphoma, with healthy individuals as controls. Multi-parameter flow cytometry using clinically validated protocols identified the main B-lymphocytes subsets (**Figure 1A**). Dimensionality reduction by t-SNE illustrated their distribution (**Figure 1B**) and enabled clear distinction between class-switched (blue) and non-class-switched (red) B- lymphocytes (**Figure 1C**), ultimately delineating at least seven distinct B-lymphocytes populations (**Figure 1D**).

Clinical stage progression was associated with profound reshaping of the B-lymphocytes landscape (**Figure 1E**). Both unswitched memory B-lymphocytes (USMBCs) and class-switched memory B-lymphocytes (SMBCs) declined progressively with advancing disease stage, while lymphoma patients exhibited a marked increase of immature and transitional B-lymphocytes (I/T-BL). Naïve B-lymphocytes also showed a generalized decline across stages, with only a transient increase in Stage 2. Together, these changes suggested a disruption of B-lymphocytes homeostasis as disease advances, with direct implications for adaptive immune competence.

Quantitative analysis confirmed these observations (**Figure 1F**, **Supplementary Table 9**). Total lymphocyte and total B-lymphocytes count declined significantly with disease progression (Kruskal-Wallis *p* < 0.05), with the steepest reductions observed in lymphoma patients. Beyond global declines, specific subsets such as naïve B lymphocytes, USMBCs, and SMBCs were significantly depleted (e.g., S1 *vs*. S3, *p* = 0.00077). Isotype-switched populations were similarly affected (e.g., S1 *vs*. S3, *p* = 2.7 × 10^-5^).

The loss of SMBCs was particularly striking, extending across multiple surface isotypes (smIgA1^+^, smIgA2^+^, smIgG1^+^, smIgG2^+^, smIgG3^+^, and smIgG4^+^). This broad depletion highlights a progressive erosion of humoral memory, most severe in the lymphoma cohort (**Figure 1F**). Such findings underscore the inability of ART to fully restore B-lymphocytes function, even under conditions of virological suppression.

Interestingly, not all B-lymphocytes populations followed a pattern of decline. A progressive expansion of I/T-BL was observed, with significant differences across clinical stages and in lymphoma patients (S1 *vs*. S2, *p=* 0.021; S1 *vs*. S3, *p=* 0.0085; S2 *vs*. lymphoma, *p=* 0.018). In parallel, SMBC-/smIgM^+^ subsets also expanded progressively with disease stage (S1 *vs*. S2, *p=* 0.013; S1 *vs*. S3, *p=* 3.8 × 10^-5^). These expansions suggest a skewing of B-lymphocytes maturation toward immature and atypical compartments.

Correlation analyses further reinforced these findings (**Figure 1F**, **Supplementary Table 10**). Clinical stage showed strong negative associations with multiple subsets, explaining declines of 41% in total lymphocytes (ρ_spearman_ = -0.41), 26% in total B-lymphocytes (ρ_spearman_ = -0.26), 22% in naïve B-lymphocytes (ρ_spearman_ = -0.22), 29% in USMBCs (ρ_spearman_ = -0.29), and 39% in SMBCs (ρ_spearman_ = -0.39). Similar patterns emerged in memory isotypes, including smIgA1 (ρ_spearman_ = -0.40), smIgA2 (ρ_spearman_ = -0.36), smIgG1 (ρ_spearman_ = -0.33), smIgG2 (ρ_spearman_ = -0.43), smIgG3 (ρ_spearman_ = -0.31), and smIgG4 (ρ_spearman_ = -0.35).

Collectively, these data demonstrate that HIV clinical progression is accompanied by a profound and progressive loss of B-lymphocytes subpopulations, particularly within the memory compartment, while favoring the expansion of immature subsets. This dual phenotype reflects an impaired capacity to sustain humoral immunity despite ART.

### 3.3 Systemic alterations in cytokine profiles associated with advanced stages and lymphoma in people living with HIV

Patients with HIV who progress to lymphoma exhibit profound systemic immune dysregulation, involving both innate and adaptive compartments and characterized by distinct cytokine signatures (21, 22). To investigate this in our cohort, we quantified plasma cytokines to determine whether disease progression and lymphoma status were associated with measurable changes in inflammatory mediators.

**Figure 2:**
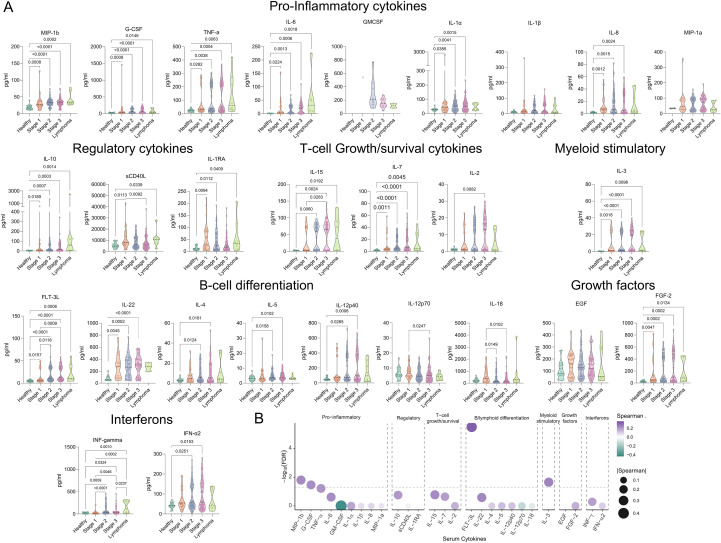
Significant changes in plasma cytokines of PLHIV according to clinical stage and their relationship with disease progression. **(A)** Plasma concentrations of 27 cytokines were quantified and categorized by biological function (pro-inflammatory, regulatory, T-lymphocytes growth/survival, myeloid-stimulatory, B-lymphocytes differentiation, growth factors, and interferons). Violin plots display cytokine levels (pg/mL) across clinical groups: healthy controls (lime green), Stage 1 (orange), Stage 2 (blue), Stage 3 (lavender), and HIV-associated lymphoma (green). **(B)** Spearman correlation analysis between clinical stage and cytokine levels. Bubble size represents the magnitude of the correlation coefficient (spearman ρ), while the y-axis shows -log_10_(p-value). Color intensity denotes the direction and strength of the correlation, and the dotted line indicates the significance threshold (p=p= 0.05). Statistical significance was determined using the Kruskal-Walli’s test (see **Supplementary Table 9**) followed by pairwise Wilcoxon comparisons.

A total of 27 cytokines were measured and classified according to their primary biological functions: pro-inflammatory, regulatory/anti-inflammatory, T-lymphocytes growth and survival, myeloid stimulatory, B-lymphocytes differentiation, growth factors, and interferons (**Figure 2**). Overall, 18 of the 27 cytokines showed statistically significant differences across clinical stages (**Supplementary Table 11**). As a general trend, patients in all disease stages exhibited higher cytokine levels compared with healthy donors, reflecting a persistent inflammatory state (**Figure 2A**).

To explore potential clinical associations, we performed correlation analyses between cytokine concentrations and clinical stage. Interestingly, no progressive cytokine escalation was observed with advancing disease stage. Only a limited subset, that includes MIP-1β, G-CSF, FLT-3L, and IL-3, displayed significant positive correlations (**Figure 2B**), suggesting that rather than a linear intensification of inflammation, people living with HIV maintain a globally dysregulated cytokine milieu throughout disease progression.

### 3.4 Progressive decline of B-lymphocytes subpopulations and chronic inflammation in PLHIV on ART coinfected with EBV

EBV coinfection is common in PLHIV and increases malignancy risk. In the setting of impaired T-cell surveillance and chronic immune activation, EBV latency/reactivation is favored, contributing to B-cell dysregulation and a sustained proinflammatory cytokine milieu (23, 24). Accordingly, we evaluated whether EBV co-infection contributes to the clinical heterogeneity observed in PLHIV.

**Figure 3:**
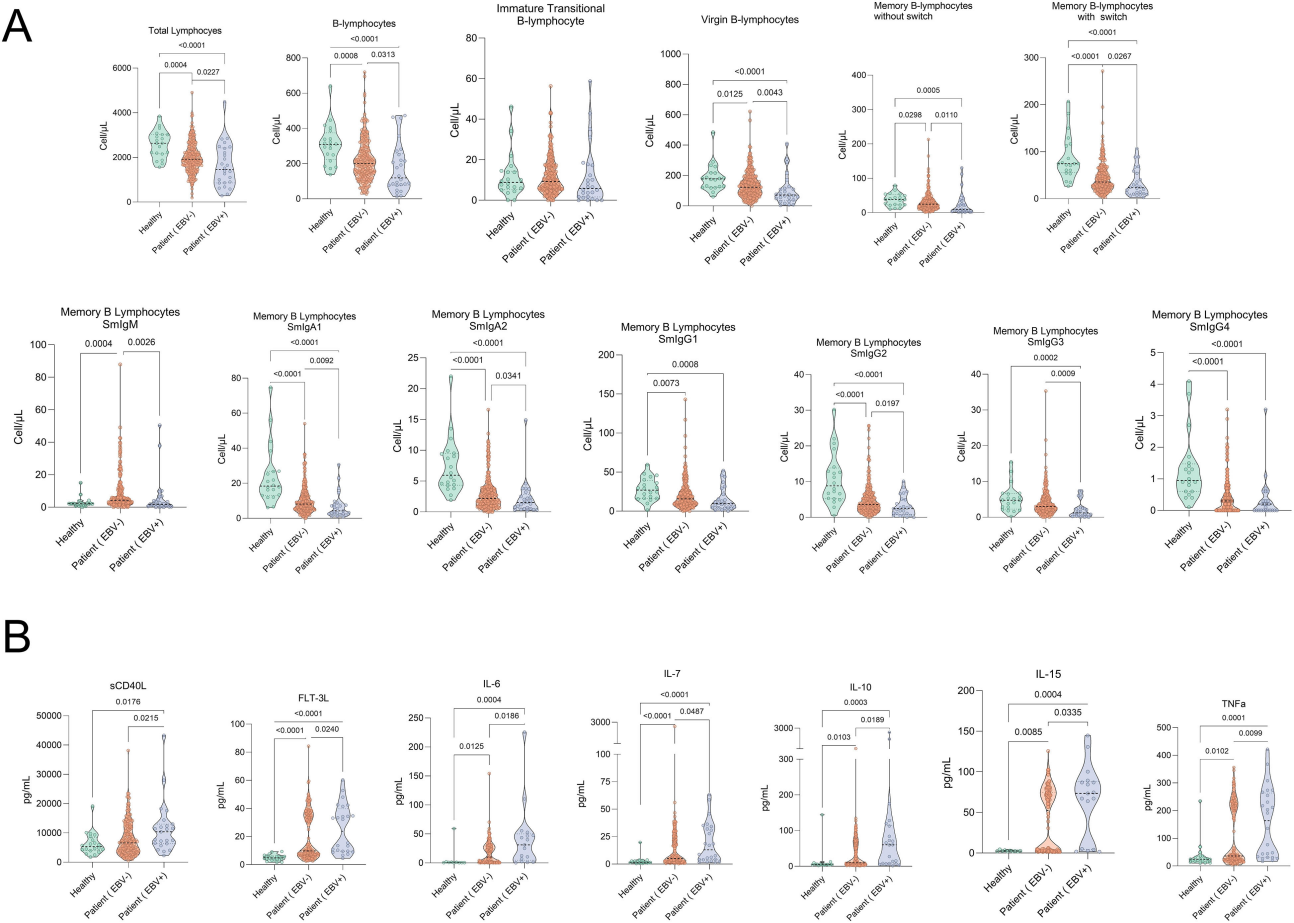
Significant comparisons of B-lymphocytes subpopulations and cytokine levels in PLHIV with or without EBV co-infection. **(A)** Comparative analysis of B-lymphocytes subpopulations in healthy controls and PLHIV without (EBV^-^) or with (EBV^+^) detectable EBV, and **(B)** plasma cytokine levels. Violin plots depict the distribution and density of each B-lymphocytes subset or cytokine (Y-axis: cells/μL or pg/mL) across three groups: healthy controls (green), PLHIV without EBV detection (EBV^-^, orange), and PLHIV with EBV detection (EBV^+^, blue). Statistical comparisons were performed using Kruskal-Walli’s test followed by Dunn’s test for multiple comparisons; exact p-values for significant differences are shown.

We classified our cohort according to EBV status (positive or negative) and compared these groups with healthy donors who presented negative viral loads (**Supplementary Table 12**). Multiple B-lymphocytes populations displayed a progressive decline from healthy controls to EBV-negative and EBV-positive individuals, including total lymphocytes, B-lymphocytes, immature/transitional B-lymphocytes, naïve B-lymphocytes, unswitched and switched memory B-lymphocytes, and surface isotype-defined subsets (smIgA1^+^, smIgA2^+^, smIgG1^+^, smIgG2^+^, smIgG3^+^, and smIgG4^+^) (**Figure 3A**). In parallel, plasma levels of several cytokines, including sCD40L, IL-6, IL-7, IL-10, IL-15, and TNF-α, showed a stepwise increase from healthy donors to EBV-negative and EBV-positive groups (**Figure 3B**). This pattern indicates a persistent pro-inflammatory milieu and sustained immune activation associated with EBV co-infection, which may contribute to ongoing immunological deterioration despite suppressive antiretroviral therapy.

### 3.5 B-lymphocytes populations remodeling associated with AIDS-defining diseases, non-AIDS conditions, and coinfections in PLHIV

Although antiretroviral therapy (ART) has significantly reduced mortality associated with AIDS-defining diseases, non-AIDS-defining diseases have become increasingly relevant causes of morbidity and mortality in PLHIV, potentially driven by persistent immune activation and chronic inflammation (25). To investigate whether these clinical variables were associated with alterations in the B-lymphocyte compartment, we stratified our cohort into four categories: (1) PLHIV without AIDS-defining diseases, non-AIDS-defining diseases, or coinfections (CI), (2) PLHIV with CI, (3) PLHIV with only AIDS-defining diseases, and (4) PLHIV with only non-AIDS-defining diseases (**Supplementary Table 13**).

**Figure 4:**
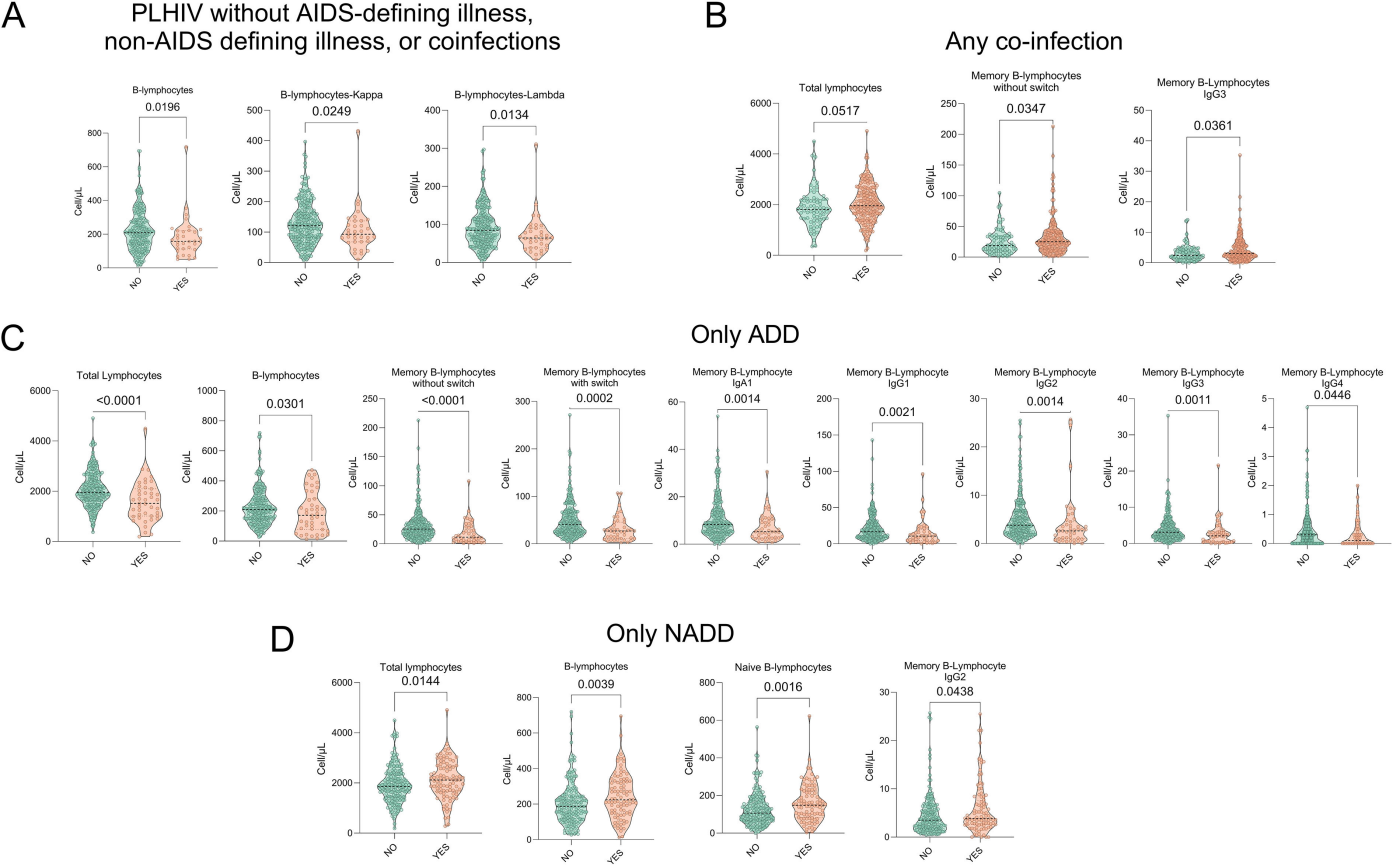
Significant comparisons of B-lymphocytes populations associated with AIDS-defining diseases, non-AIDS conditions, and coinfections in PLHIV. **(A)** PLHIV without AIDS-defining diseases, non-AIDS-defining diseases, or coinfections (CI) (YES, n=34) versus PLHIV with any clinical category (NO, n=235). **(B)** PLHIV with co-infections (YES, n=191) versus those without CI (NO, n=78) **(C)** PLHIV with ADD (YES, n=43) versus PLHIV without ADD (NO, n= 226) **(D)** PLHIV with NADD (YES, n=92) versus PLHIV without NADD (NO, n=177). Violin plots depict the distribution and density of B-lymphocytes subpopulations (Y-axis: cells/μL) across clinical groups. The median is shown as a black line within each plot. Green identifies patients without the evaluated clinical condition, while orange represents those with the condition. Statistical comparisons were performed using the Mann-Whitney test; exact p-values are shown for significant differences.

In the analysis of B lymphocyte subpopulations, it was observed that PLHIV without AIDS-defining diseases, non-AIDS-defining diseases, or coinfections (CI) presented a significant decrease in the total number of BL (**Figure 4A****).** From this observation, the other clinical categories were evaluated, finding that, in contrast, PLHIV with IC showed an increase in the total number of lymphocytes, USMBCs and SMBCs-smIgG1 +, SMBCs-smIgG3 + (**Figure 4B****).**

In patients with only AIDS-defining diseases (**Figure 4C**), we observed the most pronounced alterations in B-lymphocytes homeostasis, characterized by a broad reduction across multiple subpopulations, including total lymphocytes, total B lymphocytes, unswitched memory B-lymphocytes (USMBCs), switched memory B-lymphocytes (SMBCs), and isotype-defined subsets (smIgA1+, smIgG1+, smIgG2+, smIgG3+, and smIgG4+).

Conversely, PLHIV with only non-AIDS-defining diseases exhibited a selective increase in total lymphocytes, B lymphocytes, naïve B lymphocytes, and SMBCs-smIgG2+ (**Figure 4D**). Notably, the analysis of serum cytokine concentrations across these categories revealed no major changes (**Supplementary Table 14**), suggesting that the most significant impairment of B-lymphocytes homeostasis remains associated with the presence of AIDS-defining diseases despite ART-mediated viral suppression.

### 3.6 Loss of B-lymphocytes subpopulations and persistence of systemic inflammation in PLHIV during the early phase of antiretroviral therapy

The early phase of antiretroviral therapy (ART) represents a pivotal window in which immune reconstitution is most dynamic. Although viral suppression is typically achieved within the first months of therapy, persistent aberrations in the architecture of the immune system, especially within the B-lymphocyte compartment, often endure and may constrain long-term recovery. Indeed, even during early ART, B-lymphocytes phenotypic and functional defects present at baseline may fail to fully reverse, diminishing the capacity for robust humoral memory regeneration (7).

**Figure 5:**
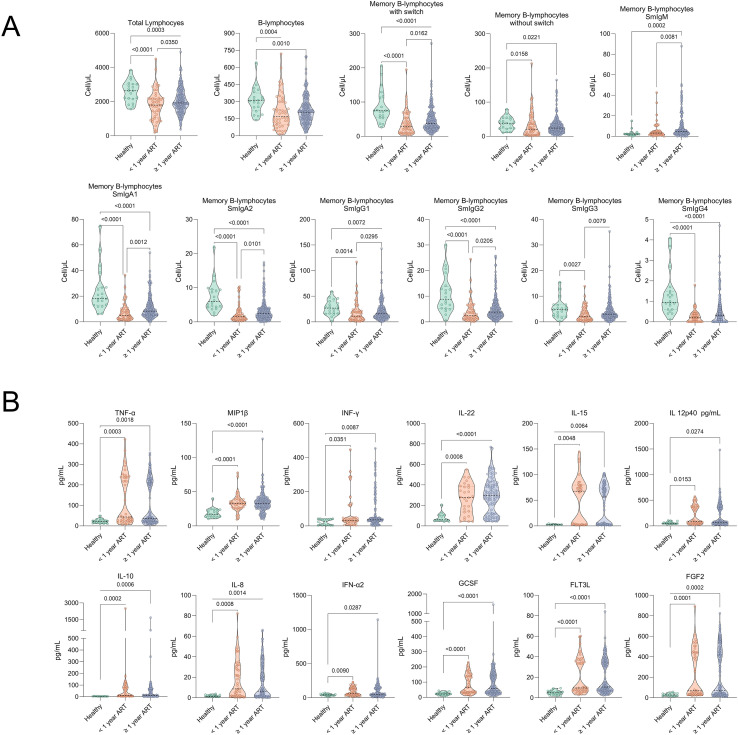
Significant alterations in B-lymphocyte subpopulations and cytokine profiles according to ART duration in PLHIV. **(A)** Comparative analysis of B-lymphocyte subpopulations in healthy controls, PLHIV on ART for less than one year (<1 year ART), and PLHIV on ART for one year or longer (≥1 year ART). **(B)** Comparative analysis of plasma cytokine levels. Violin plots represent the distribution and density of B-lymphocyte subpopulations or cytokines across three groups: healthy controls (green), PLHIV on ART <1 year (orange), and PLHIV on ART ≥1 year (blue). Each plot shows the median (black line). Statistical significance was determined by the Kruskal-Wallis test with multiple comparisons, exact p-values are shown).

To assess whether ART duration affects B-cell homeostasis and systemic inflammation, we compared healthy donors with PLHIV receiving ART for <1 year versus ≥1 year (**Figure 5****;** **Supplementary Table 15**). Both ART-treated groups showed significant reductions in multiple B-cell subsets, including total B-cells and memory B-cells (switched and unswitched) and several isotype-defined populations, relative to healthy controls (**Figure 5A**), underscoring the sustained impact of HIV on B-cell homeostasis. Participants on ≥1 year of ART exhibited modest but significant recovery in select subsets compared with <1 year, suggesting gradual, partial reconstitution with prolonged therapy (**Figure 5A**).

In parallel, plasma analysis revealed sustained elevations in several proinflammatory cytokines in both ART groups relative to healthy donors (**Figure 5B**). However, no major differences were detected between short- and long-term ART, indicating that extended viral suppression exerts limited effects on systemic cytokine normalization. Collectively, these findings suggest that early immune reconstitution is marked by persistent loss of humoral memory and chronic systemic inflammation, which may persist despite prolonged ART and be associated with long-term immune dysfunction and a higher comorbidity burden.

## 4 Discussion

The HIV epidemic exerts a disparate impact across genders and key populations. Men account for a substantial proportion of new infections and often face barriers to diagnosis and treatment, magnifying the risk of disease progression (26). Socially marginalized groups, including men who have sex with men, sex workers, and people who inject drugs, continue to bear a disproportionate burden in transmission dynamics and disease prevalence, underscoring the need for stigma-reducing, targeted interventions and expanded access to care (27, 28).

In our cohort, five patients were diagnosed with lymphoma: two with diffuse large B-cells lymphoma (DLBCL), one Burkitt lymphoma (BLT), three with Hodgkin lymphoma (HL), and two with plasmablastic lymphoma. This distribution aligns with published patterns in people living with HIV (PLHIV), in whom non-Hodgkin lymphomas (NHLs), particularly systemic DLBCL, predominate (29, 30). A limitation of this study is the age difference between healthy controls and lymphoma patients, with lymphoma patients being older on average (**Supplementary Table 5**), which reflects the known epidemiology of lymphoma (31). Because age may influence immune parameters, additional analyses adjusting for age were performed. Importantly, the age-adjusted results were consistent with the unadjusted findings (**Supplementary Table 6**), supporting the robustness of the observed differences and indicating that the immune alterations identified are not solely attributable to age. In addition, Epstein-Barr virus (EBV) is detected in roughly 30-50% of HIV-related DLBCLs, which is higher than in the general population (29, 30), and no consistent differences in age or sex have been observed between EBV-positive and EBV-negative DLBCL among PLHIV (29, 32). Mechanistically, HIV may contribute to lymphomagenesis via persistent antigenic stimulation and inflammatory signaling: tumor infiltration by CD8^+^ cytotoxic T lymphocytes has been reported in DLBCL expressing LMP1 and HIV-1 p24, a marker of active replication, while the HIV-1 matrix protein p17 may persist despite ART, acting as a cytokine-like molecule that promotes T-lymphocytes activation, angiogenesis, and upregulation of LMP1 in EBV-infected primary B-lymphocytes (29, 33, 34). These observations are consistent with recent syntheses of HIV-associated lymphomas and EBV-driven lymphomagenesis (35–37).

Our findings indicate that B-cell alterations in patients with HIV infection are not simply the result of quantitative immunodeficiency but rather reflect a dynamic and persistent immune dysregulation that persists despite antiretroviral therapy. These alterations are most pronounced in patients with aggressive B-cell lymphomas and HIV-associated Hodgkin lymphoma, likely due to tumor-specific molecular heterogeneity and microenvironmental influences (38). Although biologically distinct, these lymphomas share a common feature: the direct and indirect contribution of HIV to their pathogenesis, often in cooperation with the Epstein-Barr virus (EBV). HIV proteins such as gp120 and Nef can promote B-cell activation, immunoglobulin class switching, and AID expression, thereby increasing polyclonal activation and genomic instability that favor lymphomagenesis (39). Persistent antigenic stimulation driven by HIV and EBV, increased survival factors such as BAFF, and disrupted interactions between Tfh and B cells further compromise the effective generation of memory B cells (39). As a compensatory response, the bone marrow increases the release of immature or transitional B cells; however, in a chronic inflammatory environment, this regenerative attempt promotes sustained activation rather than proper maturation into long-lasting functional memory B cell populations (39). Overall, alterations in the B cell compartment in HIV-positive lymphoma patients result from the interplay between chronic immune dysregulation (even under antiretroviral therapy), viral cooperation, and tumor heterogeneity, which together determine the biology of the disease and clinical outcomes. Pro-inflammatory cytokines such as TNF-α, IL-6, and IFN-γ, along with IL-1β, IL-10, and IL-12, have been interrogated to characterize the immune response during hyperacute HIV-1 subtype C infection and its relationship to inflammatory pathobiology and disease progression (40). TNF-α and IL-6 are elevated in HIV and associate with immune activation, disease progression, and inflammatory pathology. IFN-γ supports antiviral defense but can also contribute to immunopathology, underscoring its context-dependent role in HIV (40). Additional work evaluating cytokine levels against plasma HIV RNA shows that while IL-12, IL-15, TNF-α, and FGF-2 are higher without virologic control, they may not track directly with viremia; notably, MIP-1β can be inversely associated with viral load, suggesting distinct regulatory dynamics (41). Although it might be expected that a greater number of cytokines would correlate with clinical stage, our results are consistent with current evidence indicating that, in people living with HIV (PLWH), only a specific subset of cytokines directly reflects clinical progression, whereas many others remain persistently altered regardless of stage due to residual chronic immune activation even under antiretroviral therapy (42).

The cytokines that showed significant associations in our study (MIP-1β, G-CSF, FLT-3L, and IL-3) play key roles in cellular recruitment and hematopoietic regulation. MIP-1β participates in immune cell recruitment and reflects the persistent inflammatory activation characteristic of HIV infection (43). G-CSF, FLT-3L, and IL-3 regulate the proliferation and differentiation of hematopoietic progenitors, including B-cell and dendritic cell precursors, and their dysregulation has been associated with immune dysfunction, disruption of lymphoid homeostasis, and an increased risk of hematologic abnormalities in patients living with HIV (3).

Taken together, these findings suggest that cytokines directly involved in hematopoietic regulation and lymphoid compartment homeostasis may be more sensitive indicators of clinical progression, whereas other inflammatory cytokines remain relatively elevated in a more constant manner. which, broadly align with reports of residual inflammation on suppressive ART and its clinical consequences, including non-AIDS comorbidities (44). Within the B-lymphocyte compartment, HIV infection perturbs immune homeostasis at multiple levels. Elevated frequencies of immature and transitional B-lymphocytes have been associated with CD4+ lymphopenia, implicating impaired early B-lymphocytes maturation; IL-7 may act in a compensatory, homeostatic fashion in this setting (5).

PLHIV also exhibit expansion of exhausted/hyperactivated mature B-lymphocytes and increased short-lived plasmablasts, consistent with polyclonal activation and dysregulated humoral maturation (6, 7). Longitudinal data indicate that while ART partially restores certain B-lymphocyte subsets, normalization is incomplete relative to healthy donors: transitional B-lymphocytes and plasmablasts often remain elevated, and naïve B-lymphocytes reduced, even with therapy; nonetheless, ART is associated with higher naïve B-lymphocytes frequencies compared with acute infection, highlighting a partial but insufficient recovery (8).

The incomplete recovery of the B-cell compartment and the persistent skewing toward immature phenotypes despite ART reflect complex mechanisms associated with HIV pathogenesis and chronic immune dysfunction (45). Although ART effectively suppresses viral replication, alterations in B-cell distribution persist, including increased frequencies of transitional and immature B cells together with a sustained state of immune activation and exhaustion (46).

Underlying mechanisms include impaired interactions between B cells and follicular helper T (Tfh) cells, which are essential for B-cell maturation and memory maintenance; residual inflammation and chronic immune activation, which promote the expansion of dysfunctional subpopulations; and alterations in B-cell receptor repertoire diversity and selection, which may limit effective humoral responses (10, 45).

These alterations contribute to long-term immune dysfunction and increased susceptibility to infections, even in patients with prolonged viral suppression (47). Our findings reinforce the evidence that HIV infection induces profound and persistent changes in the B-cell compartment that underlie chronic immune dysregulation.

Abnormal immunoglobulin subclass profiles are another hallmark of HIV-associated humoral dysregulation (48). In our cohort, IgM was increased across stages 1-3, whereas IgD and IgG4 were nearly absent (with significant differences for IgG4 in stages 2–3 *vs*. controls). IgA1, IgA2, IgG1, and IgG2 declined progressively with advancing disease; additional decrements were observed for IgG2 (stage 2 *vs*. 3) and IgG3 (stage 3 *vs*. controls). These patterns point to defective class-switch recombination and memory attrition. These immunoglobulin disturbances have been described as clinically relevant given their association with impaired vaccine responsiveness and increased risk of certain infections (49).

Finally, comparisons among elite controllers (ECs), patients on combination ART (cART), and HIV-negative donors illustrate distinct B-lymphocytes dynamics. ECs may show slightly higher total B-lymphocytes proportions *vs*. HIV-negative donors (often not statistically significant), with similar memory distributions across activated, intermediate, and resting states, but increased tissue-like memory cells relative to HIV-negative controls. HIV-specific B-lymphocytes in ECs predominantly express IgG1, with occasional IgG2/IgG3; antigen-specific antibody-secreting responses are enriched in ECs and rare in cART recipients, suggesting qualitatively distinct humoral responses in natural controllers (50).

By profiling B-cell subsets, cytokines, and EBV across clinical stages, we identify several clinically relevant patterns. First, we observe a stage-linked loss of memory B cells expressing switched isotypes (smIgA1/2 and smIgG1-4) despite ART, with the most severe deficits in patients with lymphoma, consistent with germinal-center dysfunction and impaired Tfh-B-cell help. Second, EBV-positive individuals show deeper B-cell depletion and higher inflammatory mediators (IL-6, IL-10, IL-15, TNF-α, and sCD40L), aligning with EBV reactivation and extrafollicular B-cell activation in the setting of weakened immune surveillance. Third, early ART (<1 year) is marked by incomplete, immature-skewed B-cell reconstitution and persistent cytokine elevations, with only modest improvement after ≥1 year, suggesting that virologic suppression alone does not restore B-cell homeostasis or resolve systemic inflammation.

The composition of plasmablasts (PBs) in people living with HIV (PLWH) has been associated with an increased proportion of circulating PBs during the early stages of infection; however, only a small fraction of these PBs are HIV-specific. Antiretroviral therapy (ART) has been shown to reduce both total and HIV-specific PBs in infected individuals (6).

On the other hand, in patients with high levels of EBV viral load in peripheral blood, the presence of the virus has been detected in circulating PBs and plasma cells in approximately half of the cases, challenging the previous concept that EBV was exclusively restricted to B cells at earlier stages of differentiation (51). Additionally, it has been reported that 10–20% of EBV-carrying plasma cells may undergo lytic replication, suggesting that terminal differentiation into plasma cells is a key step for viral reactivation (52).

Together, these findings delineate an integrated immunological signature of incomplete reconstitution under ART, providing mechanistic insight into the persistent vulnerability of PLHIV to inflammatory comorbidities and lymphoma. From a translational perspective, these results highlight the value of including advanced immune profiling in HIV care to detect subclinical immune dysregulation and to guide preventive strategies, including vaccination timing and early identification of patients at higher immunological risk.

## 5 Conclusion

The findings of this study demonstrate that, despite effective viral suppression achieved with ART, people living with HIV experience incomplete immune reconstitution characterized by a profound and progressive loss of humoral memory, expansion of immature B cells, and a persistent proinflammatory milieu. These alterations, often exacerbated in advanced clinical stages, in the presence of EBV co-infection, and further modulated by treatment duration and comorbid conditions, create a state of immune dysfunction that promotes both clinical progression and the development of non-AIDS-defining diseases and HIV-associated lymphomas. Collectively, our results underscore the need to integrate standardized immunological monitoring beyond CD4+ T-cell counts and viral load into clinical practice, to improve prevention, surveillance, and personalized patient care.

## Data Availability

The original contributions presented in the study are included in the article/**Supplementary Material**. Further inquiries can be directed to the corresponding author.
