# Supplementary material for: Incomplete B-cell reconstitution in ART-treated people living with HIV is associated with EBV-linked lymphoma progression
Source: Front Immunol. 2026 Mar 18;17:1791480. doi: 10.3389/fimmu.2026.1791480 (PMC13038446; doi:10.3389/fimmu.2026.1791480)
Supplement: Supplementary file 2 [file Table1.docx]

Supplementary Material

**Table S1. Classification of clinical stages according to the CDC and distribution of study participants (1).**

|  | N | Description at diagnosis |
| --- | --- | --- |
| Stage 1 | 72 | Asymptomatic; no signs of immunodeficiency; >500 CD4⁺ T cells/mm³ (CDC) |
| Stage 2 | 100 | Mild symptoms; moderate immunodeficiency; 200–499 CD4⁺ T cells/mm³ (CDC) |
| Stage 3 | 92 | Severe immunodeficiency; <200 CD4⁺ T cells/mm³ (CDC) |
| Recently diagnosed B-cell lymphoma | 8 | Patients with a recent diagnosis of B-cell lymphoma |
| Healthy controls | 20 | Individuals without HIV diagnosis and without immunodeficiency |

N, Number of participants in the study.

**Table S2. Clinical coinfections recorded in PLHIV are included in the study.**

| Type of coinfection, n= 194 | |
| --- | --- |
| Syphilis, n (%) | 127 (65.5) |
| Latent tuberculosis, n (%) | 6 (3.1) |
| Varicella-zoster virus (VZV), n (%) | 12 (6.2) |
| Human papillomavirus (HPV), n (%) | 68 (35.1) |
| Herpes simplex virus (HSV), n (%) | 11 (5.7) |
| SARS-CoV-2 (COVID-19), n (%) | 28 (14.4) |
| Monkeypox virus (MPXV), n (%) | 11 (5.7) |
| Molluscum contagiosum, n (%) | 3 (1.5) |
| Proctitis, n (%) | 12 (6.2) |
| Cytomegalovirus (CMV), n (%) | 5 (2.6) |
| Hepatitis C virus (HCV), n (%) | 33 (17) |
| Hepatitis B virus (HBV), n (%) | 16 (8.2) |
| *Neisseria gonorrhoeae (*urethritis/gonococcal infection*), n (%)* | 12 (6.2) |
| *Helicobacter pylori (H. pylori), n (%)* | 4 (2.1) |
| Candidiasis, n (%) | 3 (1.5) |
| Meningitis, n (%) | 1 (0.5) |
| Hepatitis A virus (HAV), n (%) | 1 (0.5) |

n, number of participants; %, percentage. * Participants could present more than one coinfection.

**Table S3. AIDS-Defining Illness recorded in the medical history of PLHIV included in the study (2).**

| Type n= 44 | |
| --- | --- |
| Esophageal candidiasis, n (%) | 9 (20.5) |
| Extrapulmonary cryptococcosis, n (%) | 4 (9.1) |
| Herpes simplex: chronic ulcers (>1 month of duration) or bronchitis, pneumonitis, or esophagitis (>1 month of duration), n (%) | 5 (11.4) |
| Disseminated or extrapulmonary histoplasmosis, n (%) | 6 (13.6) |
| Multiple or recurrent bacterial infections, n (%) | 6 (13.6) |
| Burkitt’s lymphoma (or equivalent term), n (%) | 2 (4.5) |
| Immunoblastic lymphoma (or equivalent term), n (%) | 3 (6.8) |
| *Mycobacterium tuberculosis* (any site: pulmonary, extrapulmonary, or disseminated)*, n (%)* | 16 (36.4) |
| *Pneumocystis jirovecii* pneumonia, n (%) | 4 (9.1) |
| Cerebral toxoplasmosis (>1 month of duration), n (%) | 5 (2.6) |
| HIV-associated wasting syndrome, n (%) | 16 (36.4) |
| Diffuse large B-cell lymphoma, n (%) | 2 (4.5) |
| Bronchial, tracheal, or pulmonary candidiasis, n (%) | 1 (2.3) |
| Cytomegalovirus disease (affecting liver, spleen, or lymph nodes; >1 month of duration), n (%) | 5 (2.6) |
| Recurrent pneumonia, n (%) | 1 (2.3) |
| *Mycobacterium avium* complex or *Mycobacterium kansasii* (disseminated or extrapulmonary), n (%) | 1 (2.3) |

n, number of participants; %, percentage. *Participants could present more than one coinfection.

**Table S4. Non-AIDS-Defining illness clinically recorded in PLWH included in the study.**

| Types, n= 95 | |
| --- | --- |
| Hypertension (HTN), n (%) | 19 (20) |
| Obesity, n (%) | 16 (16.8) |
| Diabetes mellitus , n (%) | 3 (3.2) |
| Coronary heart disease, n (%) | 1 (1.1) |
| Liver disease, n (%) | 11 (11.6) |
| Hypothyroidism, n (%) | 29 (30.5) |
| Dyslipidemia, n (%) | 47 (49.5) |
| Chronic kidney disease, n (%) | 3 (3.2) |
| Osteopenia, n (%) | 5 (5.3) |

n, number of participants; %, percentage. *Participants could present more than one non-AIDS-defining illness.

**Tabla S5. Demographic characteristics of healthy controls and patients with lymphoma**

| Variable | Healthy controls  (n=20) | Lymphoma  (n=8) | p-value |
| --- | --- | --- | --- |
| Age, median (IQR), years | 33 (26.25–40.5) | 47 (38.0–55.25) | 0.022 |
| Sex, n (%) |  |  |  |
| — Male | 16 (80%) | 8 (100%) | ns |
| — Female | 4 (20%) | 0 (0%) |  |

*Mann-Whitney U test for age; Fisher's exact test for sex.*

**Table S6. Comparisons of B-cell subsets between healthy controls and lymphoma patients after adjustment for age and multiple-testing correction.**

| **Variable** | **Kruskal-Wallis** | **Healthy vs Lymphoma**  **(unadjusted p-value)** | **Healthy vs Lymphoma**  **(age-adjusted p-value)** | **Age-adjusted p-value after multiple-testing correction (FDR/BH)** |
| --- | --- | --- | --- | --- |
| B-cell (Cell/μL) |  |  |  |  |
| BL | 3.628E-05 | 2.586E-02 | 3.806E-04 | 2.561E-03 |
| BL- Kappa | 2.963E-05 | 1.688E-02 | 1.060E-04 | 1.046E-03 |
| BL-Lambda | 7.780E-05 | 2.586E-02 | 2.114E-03 | 1.079E-02 |
| I/T - BL | 1.180E-02 | 8.325E-01 | 1.665E-01 | 2.933E-01 |
| I/T - BL Kappa | 1.134E-02 | 5.923E-01 | 1.552E-01 | 2.802E-01 |
| I/T - BL Lambda | 7.105E-03 | 1.234E-01 | 7.989E-02 | 1.902E-01 |
| Naive BL | 5.943E-04 | 2.097E-02 | 8.153E-03 | 3.549E-02 |
| Relation K/L-BL | 3.986E-01 |  | 1.311E-01 | 2.521E-01 |
| SMBCs | 6.492E-10 | 2.896E-04 | 9.381E-06 | 1.388E-04 |
| SMBCs-smIgA1 | 1.181E-10 | 1.930E-04 | 1.816E-07 | 5.374E-06 |
| SMBCs-smIgA2 | 7.143E-10 | 2.033E-03 | 8.481E-07 | 1.850E-05 |
| SMBCs-smIgD | 3.186E-01 |  | 7.673E-01 | 8.539E-01 |
| SMBCs-smIgG1 | 1.591E-06 | 2.097E-02 | 2.984E-02 | 9.397E-02 |
| SMBCs-smIgG2 | 3.491E-11 | 8.751E-04 | 6.786E-06 | 1.116E-04 |
| SMBCs-smIgG3 | 4.483E-06 | 4.391E-02 | 4.564E-02 | 1.228E-01 |
| SMBCs-smIgG4 | 3.734E-09 | 1.066E-03 | 8.752E-07 | 1.850E-05 |
| SMBCs-smIgM | 2.589E-06 | 1.000E+00 | 4.173E-01 | 5.825E-01 |
| Total Lymphocytes | 3.674E-10 | 3.173E-02 | 8.720E-05 | 1.046E-03 |
| USMBCs | 3.761E-06 | 1.239E-02 | 3.744E-02 | 1.074E-01 |

*Age correction was performed using generalized linear models including age as a covariate, followed by Benjamini–Hochberg multiple-testing correction..*

**Table S7. Antibody panel used for the immunophenotyping of B-lymphocyte subpopulations.**

**(3).**

|  | BV 421 | BV 510 | BV 711 | BV 605 | BV 786 | FITC | | PE | PerCP-Cy5.5 | APC | | PECy7 | APC R700 | APC-H7 |
| --- | --- | --- | --- | --- | --- | --- | --- | --- | --- | --- | --- | --- | --- | --- |
| Marker | CD27 | smIgM | CD21 | CD24 | CD19 | smIgG3+ smIgG2 | | smIgG1+  smIgG2 | smIgA1+  smIgA2 | smIgG4 +  smIgA1 | | CD5 | CD38 | smIgD |
| Antibody Clone | M-T271 | MHM-88 | B-LY4 | ML5 | SJ25C1 | SAG3/  SAG2 | SAG1/ SAG2 | | SAA1/  SAA2 | | SAG4/  SAA1 | L17F12 | HB7 | IA6-2 |
| Volume | 5 uL | 5 uL | 5 uL | 5 uL | 5 uL | 25 uL | | | | | | 5 uL | 5 uL | 5 uL |
| Catalog number (BD) | 562514 | 563113 | 563163 | 562788 | 563325 | CYT-IGS-1 | | | | | | 348810 | 659118 | 561305 |

BV, BrilliantViolet; FITC, fluorescein isothiocyanate; PE, phycoerythrin; PerCP-Cy5.5, peridinin chlorophyll protein-cyanine 5.5; APC, allophycocyanin; PECy7, phycoerythrin-cyanine 7; APC-R700, allophycocyanin-Cyanine 7; R700, Alexa Fluor 700.

**Table S8. Panel of antibodies used to identify the expression of kappa and lambda light chains in B lymphocytes.**

|  | **FITC** | **PE** | **PerCP-Cy5·5** | **PE-Cy7a** | **APC** | **APC H7** | **BV450** | **V500** |
| --- | --- | --- | --- | --- | --- | --- | --- | --- |
| **Marker** | CD8 | CD56 | CD5 | CD19 | CD3 | CD38 | CD20 | CD45 |
|  | Lambda | Kappa |  | TCR γ /δ |  |  | CD4 |  |
| **Antibody Clone** | SK1 (Leu-2a) | MY31 (Leu-19) | L17F12 | SJ25-C1 | SK7 | HB7 | L27 | 2Dl (anti-HLe-1) |
|  | 1-155-2 | TB28-2 |  | 11F2 |  |  | SK3 (Leu-3a) |  |
| **Catalog number (BD)** | 658619 | | | | | | | |

FITC, fluorescein isothiocyanate; PE, phycoerythrin; PerCP-Cy5·5, peridinin chlorophyll-cyanine 5·5; PECY7, phycoerythrin-Cyanine 7; APC, allophycocyanin; BV, Brilliant Violet.

**Table S16. Distribución de plasmablastos en las categorías clínicas.**

| Población | Categoría | *p-*valor |
| --- | --- | --- |
| - Total plasma cells | Lymphoma vs Stage 1  Lymphoma vs Stage 2  Lymphoma vs Stage 3  Stage 1 vs Stage 2  Stage 1 vs Stage 3  Stage 2 vs Stage 3  Without ADD, NADD, CI  Only ADD (No vrs Yes)  Only NADD (No vrs Yes)  Only CI (No vrs Yes)  EBV (Control vrs Positive)  EBV (Control vrs Negative)  EBV (Negative vrs Positive)  TAR <1 año vrs >1 año | 0.68  0.83  0.78  0.68  0.78  0.86  0.45  0.86  0.19  0.46  0.01  0.08  0.05  0.06 |
| - Plasma cells Kappa | Lymphoma vs Stage 1  Lymphoma vs Stage 2  Lymphoma vs Stage 3  Stage 1 vs Stage 2  Stage 1 vs Stage 3  Stage 2 vs Stage 3  Without ADD, NADD, CI  Only ADD (No vrs Yes)  Only NADD (No vrs Yes)  Only CI (No vrs Yes)  EBV (Control vrs Positive)  EBV (Control vrs Negative)  EBV (Negative vrs Positive)  TAR <1 año vrs >1 año | 0.53  0.76  0.76  0.53  0.71  0.80  0.22  0.93  0.28  0.58  0.01  0.08  0.05  0.08 |
| - Plasma cells Lambda | Lymphoma vs Stage 1  Lymphoma vs Stage 2  Lymphoma vs Stage 3  Stage 1 vs Stage 2  Stage 1 vs Stage 3  Stage 2 vs Stage 3  Without ADD, NADD, CI  Only ADD (No vrs Yes)  Only NADD (No vrs Yes)  Only CI (No vrs Yes)  EBV (Control vrs Positive)  EBV (Control vrs Negative)  EBV (Negative vrs Positive)TAR <1 año vrs >1 año | 0.86  0.84  0.79  0.89  0.85  0.92  0.85  0.73  0.16  0.32  0.01  0.10  0.05  0.07 |

# Supplementary Figure 1

**
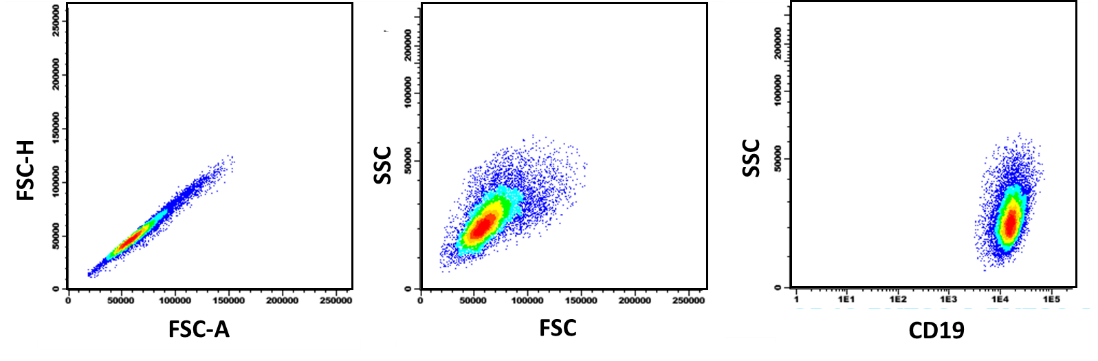
A**

**
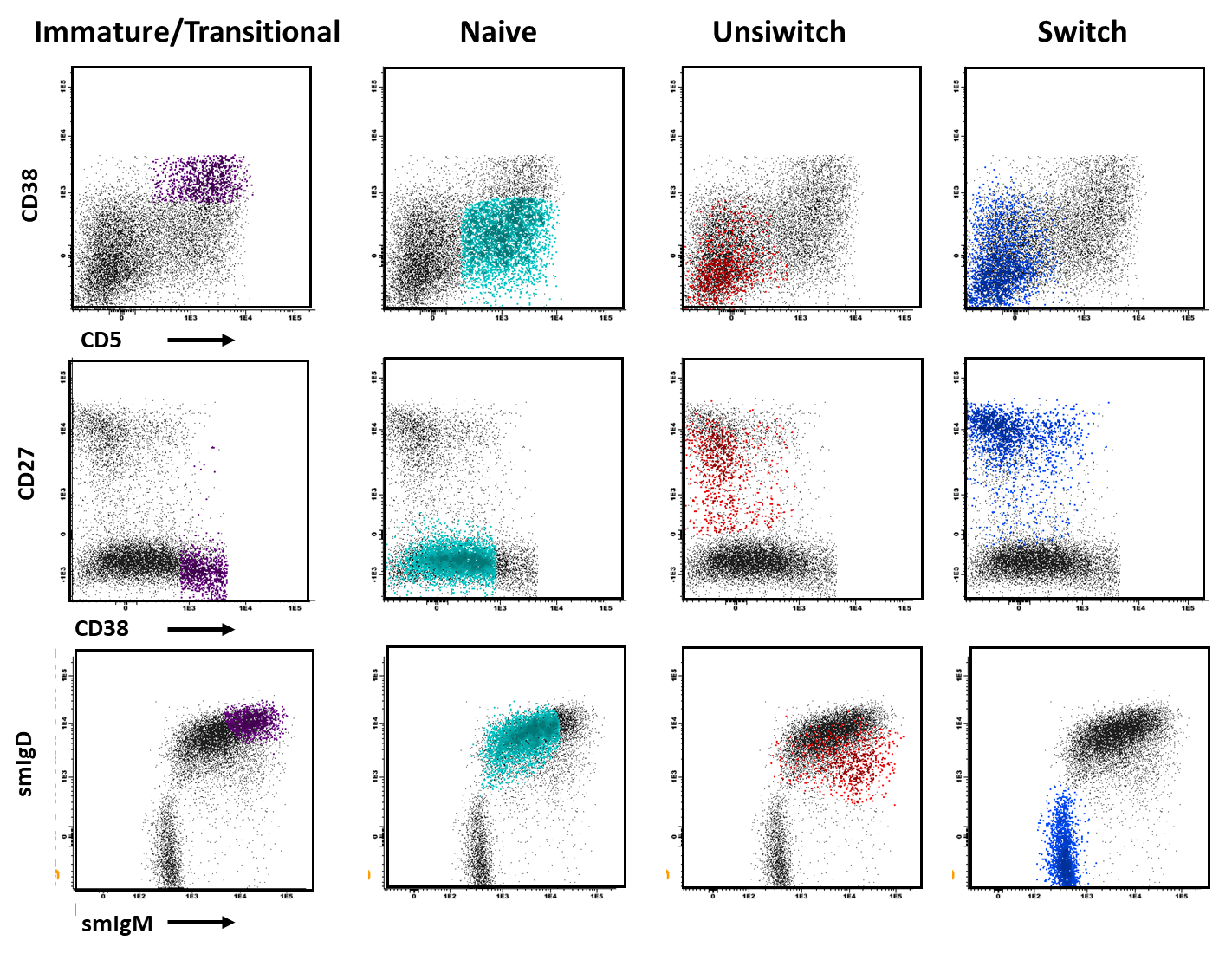
B**

**
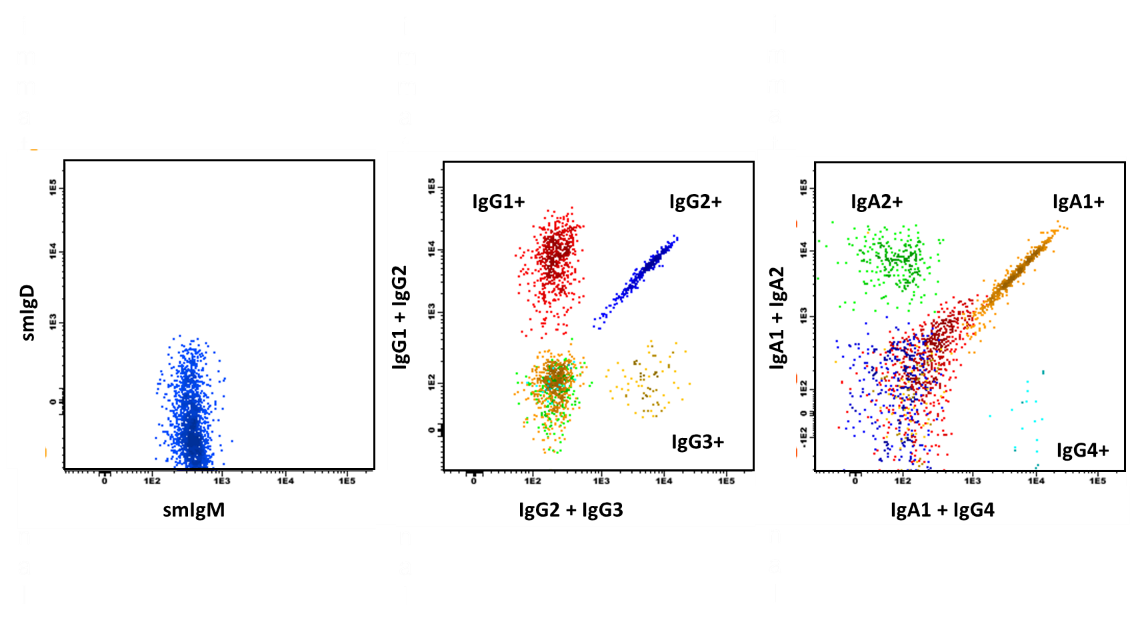
**

**C**

**Supplementary Figure 1. Analysis strategy of circulating B cells. A. Identification of B cells FSC/SSC^lo^ CD19^+^. B Immature/transitional B cells (CD5^+^ CD27^−^ CD38^++^ smIgM^++^ smIgD^+^) shown in purple, CD5^+^ naïve B cells (CD27^−^ CD38^+^ smIgM^+^ smIgD^++^) shown in cyan, and memory B cells without and with isotype switching (CD5^−^ CD27^+^ CD38^−^ smIgM+ smIgD^++^ and CD5^−^ CD27^+/−^ CD38^−^ smIgM^−^ smIgD^−^, respectively) shown in red and blue. C Analysis of the IgH isotype tube for the characterization of class-switched memory B cells in blood samples from healthy controls and people living with HIV. Bivariate plots show the distribution of class-switched memory B cells (smIgMD^−^CD19^+^CD38^−^) according to the surface expression of different IgH isotypes (IgG1, IgG2, IgG3, IgG4, IgA1, and IgA2).**

**Supplementary Bibliography**

1. The publication was produced for the U.S. Department of Health and Human Services (HHS) HRaSAHbtAEaTCN. guide for HIV/AIDS Clinical Care 2014 [Available from: chrome-extension://efaidnbmnnnibpcajpcglclefindmkaj/<https://ryanwhite.hrsa.gov/sites/default/files/ryanwhite/grants/2014-guide.pdf>.

2. Richard M. Selik MEDM, MPH 2 Bernard Branson, MD 1 S. Michele Owen, PhD 1 Suzanne Whitmore, DrPH 1 H. Irene Hall, PhD. Revised Surveillance Case Definition for HIV Infection — United States, 2014*Recommendations and Reports* 2014 [Available from: <https://www.cdc.gov/mmwr/preview/mmwrhtml/rr6303a1.htm>.

3. Blanco E, Pérez-Andrés M, Arriba-Méndez S, Contreras-Sanfeliciano T, Criado I, Pelak O, et al. Age-associated distribution of normal B-cell and plasma cell subsets in peripheral blood. J Allergy Clin Immunol. 2018;141(6):2208-19.e16.
